# Rapid onset of cardiomyopathy in STZ-induced female diabetic mice involves the downregulation of pro-survival Pim-1

**DOI:** 10.1186/1475-2840-13-68

**Published:** 2014-04-01

**Authors:** Andrew Moore, Amol Shindikar, Ingrid Fomison-Nurse, Federica Riu, Pujika E Munasinghe, Thrishila Parshu Ram, Pankaj Saxena, Sean Coffey, Richard W Bunton, Ivor F Galvin, Michael JA Williams, Costanza Emanueli, Paolo Madeddu, Rajesh Katare

**Affiliations:** 1 Department of Physiology-HeartOtago, Otago School of Medical Sciences, University of Otago, PO Box 913, Dunedin 9054, New Zealand; 2 Departments of Cardiothoracic Surgery, Dunedin School of Medicine, University of Otago, Dunedin, New Zealand; 3 Departments of Medicine, Dunedin School of Medicine, University of Otago, Dunedin, New Zealand; 4 Chair of Experimental Cardiovascular Medicine, School of Clinical Sciences, Bristol Heart Institute, University of Bristol, Bristol, UK; 5 The University of Western Australia, Perth, Australia; 6 Oxford Biomedical Research Centre, Oxford, UK

**Keywords:** Diabetes, Cardiomyopathy, Gender difference, Cardiac dysfunction, Apoptosis

## Abstract

**Background:**

Diabetic women are five times more likely to develop congestive heart failure compared with two fold for men. The underlying mechanism for this gender difference is not known. Here we investigate the molecular mechanisms responsible for this *female disadvantage* and attempt safeguarding cardiomyocytes viability and function through restoration of pro-survival Pim-1.

**Methods and Results:**

Diabetes was induced by injection of streptozotocin in CD1 mice of both genders. Functional and dimensional parameters measurement using echocardiography revealed diastolic dysfunction in female diabetic mice within 8 weeks after STZ-induced diabetes. This was associated with significant downregulation of pro-survival Pim-1 and upregulation of pro-apoptotic Caspase-3, microRNA-1 and microRNA-208a. Male diabetic mice did not show any significant changes at this time point (P < 0.05 vs. female diabetic). Further, the onset of ventricular remodelling was quicker in female diabetic mice showing marked left ventricular dilation, reduced ejection fraction and poor contractility (P < 0.05 vs. male diabetic at 12 and 16 weeks of STZ-induced diabetes). Molecular analysis of samples from human diabetic hearts confirmed the results of pre-clinical studies, showing marked downregulation of Pim-1 in the female diabetic heart (P < 0.05 vs. male diabetic). Finally, in vitro restoration of Pim-1 reversed the *female disadvantage* in diabetic cardiomyocytes.

**Conclusions:**

We provide novel insights into the molecular mechanisms behind the rapid onset of cardiomyopathy in female diabetics. These results suggest the requirement for the development of gender-specific treatments for diabetic cardiomyopathy.

## Background

Diabetic cardiomyopathy, a common complication in people with diabetes, deserves special clinical attention because of its insidious onset, relatively rapid evolution from diastolic to global cardiac dysfunction, [1-3] and very poor outcome once heart failure (HF) manifests [4,5].

Cardiac complications of diabetes are prevalent in women. In fact, while non-diabetic women are relatively protected from cardiovascular disease, this advantage is lost in diabetes [6]. In the Framingham study, the reported incidence of symptomatic HF was 2.4-fold higher in men and 5.0-fold higher in women with diabetes compared to their non-diabetic peers, [7] a finding confirmed by several other studies [8-10]. Furthermore, diabetic women have significantly higher mortality after myocardial infarction than diabetic men [11]. A gender difference was also noted for pre-clinical diabetic cardiomyopathy. In a series of 100 adults (44% females) without previous evidence of heart disease, echocardiography showed the presence of diabetic cardiomyopathy in 48% of patients, with female gender being one of the strongest predictors of cardiac remodelling [12]. In another series of 80 children and adolescents with well-controlled T1D, abnormalities in left ventricular dimensions and myocardial relaxation were reported, with the girls clearly being more affected than boys [13]. The reason for this global “*female disadvantage*” in diabetes remains largely unknown. Studies in animal models helped to elucidate potential pathogenic mechanisms of cardiomyopathy, but not to explain the interaction between gender and diabetes, as most available information derives from investigations performed in male rodents [14]. Hence, a deeper understanding of gender-related targets for stratified therapy is needed.

Recent studies from us [15-17] and others [18,19] have unveiled a spectrum of molecular mechanisms involved in the development of diabetic cardiomyopathy. We hypothesize that these molecular changes would appear much earlier and develop faster in the female diabetics, leading to the rapid development of cardiac dysfunction. We also propose to exploit this new knowledge for gender-specific treatment of diabetic cardiomyopathy. To this aim, we have performed a time course study comparing male and female diabetic mice from early to late stages of cardiomyopathy. Echocardiography follow-up was coupled with investigation of molecular targets. We also verified the molecular mechanisms in biopsies from human hearts. Finally, we applied a rescue approach which demonstrates for the first time the importance of Pim-1 in gender disparity of diabetic cardiomyopathy.

## Materials and methods

### Ethics

Investigation conforms to the Guide for the Care and Use of Laboratory Animals published by the US National Institutes of Health (NIH Publication No. 85-23, revised 1996) and with approval of the Animal Ethical Committees of the University of Otago, New Zealand, British Home Office and the University of Bristol, United Kingdom. Human right atrial tissue samples were collected from the patients undergoing on-pump coronary artery bypass graft surgery after written consent which conforms with the principles outlined in the Declaration of Helsinki and was approved by the Human and Disability Ethics Committee of New Zealand.

### In vivo studies

#### Mouse model of STZ-induced diabetes

Diabetes was induced in CD1 mice of both genders (Charles River, UK and HTRU, NZ) by injection of streptozotocin (STZ; 40 mg/kg body weight i.p. per day for 5 days). Age-matched animals that received STZ-vehicle served as non-diabetic controls [17,20]. Hyperglycemia was confirmed by measuring glucose levels in blood and urine. No difference was observed between genders at any time point of the study (Additional file 1: Figure S1).

### In vivo functional measurements

#### Echocardiography

Functional and dimensional parameters were measured at 4, 8, 12, 16 and 32 weeks after STZ-induced diabetes using a high-frequency echocardiography system (Vivid E9, GE Health Sciences, New Zealand) (n = 8 mice per group). Briefly, mice were anesthetized using tribromo-ethanol (Avertin) and transferred to an imaging stage equipped with a warming pad for controlled maintenance of mouse body temperature at 37°C. Standard B mode (2D) images of the heart and pulsed Doppler images of the mitral valve inflow (to estimate the diastolic dysfunction) were acquired. Thickness of the left ventricle anterior wall (LVAW) during systole (LVAW_s_) and diastole (LVAW_d_) was measured at the level of the papillary muscles in parasternal short axis at end-systole and end-diastole. LV ejection fraction (LVEF), end systolic volume (ESV) and end diastolic volume (EDV) were determined as described earlier [15,21-24].

#### Measurement of intra-ventricular pressure

Direct measurement of intra-ventricular pressure and volume was done immediately after the echocardiographic measurements at 12, 20 and 32 week time point using a high-fidelity 1.4F transducer tipped catheter (Millar Instruments, Houston, TX, USA) as explained earlier. In brief, the catheter was inserted into the right carotid artery and slowly advanced into the heart, where its position was confirmed by the rapid deflection of the diastolic pressure wave without any change in systolic pressure. After 5 minutes stabilization period, data were collected including the heart rate (HR), LV end systolic pressure (LVESP), LV end-diastolic pressure (LVEDP) and maximal rates of LV pressure rise (dP/dt_max_) and fall (dP/dt_min_). At the end of the measurement, samples were collected from left ventricle for western blotting and RT-PCR analysis.

### TUNEL staining

Apoptosis was quantified on LV cryosections (5 μm) by the terminal deoxynucleotidyltransferase (TdT)-mediated dUTP nick-end labeling (TUNEL) technique (in situ cell death detection kit Fluorescein, Roche applied science, USA). Following treatment of slides with proteinase K (20 μg/ml, 30 min at 37°C), TUNEL assay was performed according to the manufacturer’s instruction. The same sections were then stained with DAPI to recognize nuclei and α-sarcomeric actin to recognize cardiomyocytes. Twenty fields were randomly evaluated in each section at X400 magnification. The fraction of TUNEL positive nuclei over total cardiomyocyte nuclei was then calculated [17,25].

### Western blotting

The left ventricular tissue was homogenized in ice-cold RIPA buffer and protein concentration was measured using Bradford assay. Protein samples were also isolated from the right atrial appendage of the diabetic and non-diabetic patients undergoing coronary artery bypass graft surgery for ischemic heart disease. Western blot analysis was done following separation of whole tissue (50 μg)/cell extracts (20 μg) on SDS-polyacrylamide gels. Proteins were transferred to polyvinylidene difluoride membranes (PVDF, Amersham-Pharmacia) and probed with the following antibodies: Pim-1 (Cell Signaling, 1:1000), Ser473- phospho-Akt (Cell Signaling, 1:1000), Akt (Cell Signaling, 1:1000), Bcl-2 (Cell Signaling, 1:1000), Total Caspase-3 (Cell Signaling, 1:1000), cleaved-caspase-3 (Cell Signaling, 1:1000) and Receptor interacting protein (RIP) kinase (Life Technologies, 1:1000). Actin (Cell Signaling, 1:1000) was used as loading control. For detection, secondary antibody goat anti-rabbit 680nm or anti-mouse 800 nm (both from Thermo Scientific, 1:15000) or HRP-conjugated anti-rabbit or mouse were used, followed by imaging either using the fluorescence imaging system (Odyssey) or chemiluminescence reaction (ECL, Amersham Pharmacia). Density of the bands was analyzed using Image-J (NIH, USA) software, inconsistency in the actin between the samples was normalized as described earlier [26] and data expressed in fold changes.

### RT PCR

Total RNA were extracted from either mice LV samples or human right atrial appendage using commercially available kit (Qiagen). Twenty nanograms of total RNA was reverse transcribed followed by amplification using specific primers against microRNA (miR)-1 and miR-208a. U6 was used as the internal control (all the primers and kits from Life Technologies). Of note, the homology of miR expression patterns between human and mice has been well established [27]. For quantification, the amount of miR was normalized to the amount of U6 miR using the 2 − DDCT method. Each reaction was performed in triplicate and repeated 5 times [16].

### In vitro studies

We used in vitro cardiomyocytes culture to study the effect of Pim-1 overexpression on molecular and functional targets.

### Isolation and culture of adult mouse cardiomyocytes

Cardiomyocytes were isolated from diabetic and non-diabetic mice heart of both the gender at 4, 8 and 12 weeks after STZ-induced diabetes as explained earlier [28,29]. In brief, the heart was quickly removed from the chest and retrogradely perfused at a constant pressure and at 37°C, with Ca^2+^ free KH buffer for 3 minutes, followed by enzymatic digestion with collagenase type B (0.5 mg/ml; Boehringer Manheim), collagenase type D (0.5 mg/ml; Boehringer Manheim), and protease type XIV (0.02 mg/ml; Sigma) for another 3 minutes. The heart was then perfused for another 7 minutes in KH buffer containing enzyme mix and 50 μM Ca^2+^, after which the heart was removed, cut in to small chunks and further digested in a shaker at 60 rpm for 10 minutes at 37°C in the same enzyme solution. The supernatant containing the dispersed myocytes was filtered into a sterilized tube and gently centrifuged at 500 rpm for 1 minute. The Ca^2+^ concentration was restored in a step-wise manner. The final cell pellet was suspended in minimal essential medium (MEM; Sigma Chemicals) containing 1.2 mM Ca^2+^, 2.5% preselected fetal bovine serum, and 1% penicillin-streptomycin solution (pH 7.35–7.45). Cells were finally plated in the cell culture dish coated with mouse laminin (Sigma Chemicals) depending on the type of experiment.

### Transfection with Pim-1 plasmid or anti-miR

Freshly isolated cardiomyocytes from diabetic and non-diabetic murine hearts of both genders were transfected either with human Pim-1 plasmid (8 μg/1×10^6^ cells) [15], or anti-miR-1/208 (50 nM, Life Technologies) using commercially available Lipofectamine 2000 (Life Technologies) according to the manufacturer’s instructions. Scrambled sequence was used as control for both the types of experiments. Transfection efficacy was confirmed by staining the cardiomyocytes with antibody specific to human Pim-1 plasmid (Abgent, 1:100) as described in our earlier study [15]. We achieved around 65% transfection efficacy with this technique (Additional file 1: Figure S2). After 24 hours, the medium was replaced with fresh medium and after another 48 hours culture cells were used for the experiments [15].

For western blotting, cells were seeded on a 24-well dish (1×10^6^ cardiomyocytes/well). After transfection period, cells were homogenized and the resultant proteins were used for western blotting as above.

For caspase activity assay, 5×10^3^ cells were plated on 96-well dish and underwent similar transfection and treatment procedure. At the end of treatment, equal volume of caspase assay reagent was added to the wells and incubated in dark for 30 minutes at room temperature. The luminescence was read using Promega luciferase assay system [16].

### Statistical analysis

Comparison of multiple groups at different time points was performed by two-way analysis of variance (ANOVA) with Bonferroni adjustment considering the two factors (a) diabetes and (b) gender. Two-group analysis was performed by Student’s t-test. Values were expressed as mean±SD. Probability values (P) less than 0.05 were considered significant.

## Results

### Early onset of cardiac dysfunction in female diabetic mice

#### Diastolic dysfunction

**Figure 1:**
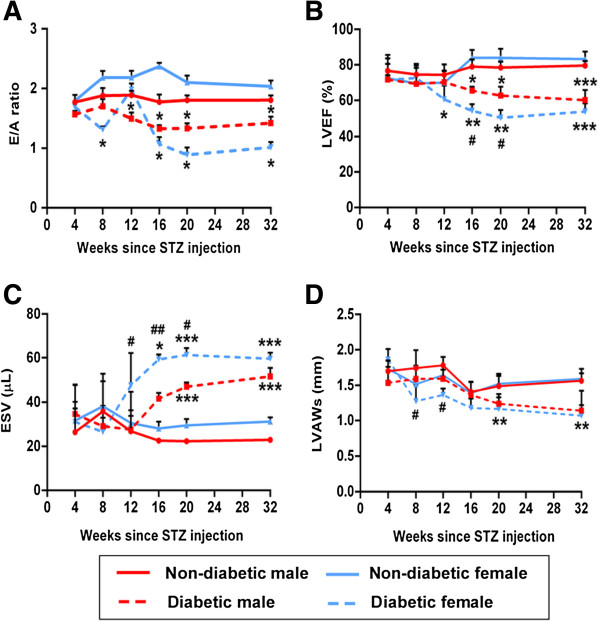
**Rapid onset of cardiomyopathy in STZ-induced female diabetic mice.** Line graphs showing the mitral valve flow velocity **(A)** and indexes of left ventricle (LV) function assessed **(B, C & D)** by the serial echocardiogram at different time points from STZ or STZ-vehicle (n = at least 8 at each time point). LVEF – LV ejection fraction, ESV – LV end systolic volume, LVAWs – LV anterior wall thickness during systole. Values are means ± SD. Results of pair-wise comparison are illustrated. *P < 0.05, **P < 0.01 and ***P < 0.001 vs. non-diabetic of corresponding gender at corresponding time point. ^#^P < 0.05 and ^##^P < 0.01 vs. male diabetic at corresponding time point.

Serial echocardiography measurement showed significant diastolic dysfunction in female diabetic mice as early as 8 weeks after STZ-induced diabetes, as evidenced by the marked decrease in the E/A ratio (Figure 1A, P < 0.05 vs. female non-diabetic). Interestingly, the E/A ratio returned to non-diabetic levels at 12 weeks. However, with progression of the disease, the index started to decline again and remained consistently low from week 16 (Figure 1A). In contrast, male diabetics did not show any changes in the E/A ratio until 12 weeks after STZ-induced diabetes (Figure 1A). Although there was no significant difference between male and female diabetics at any corresponding time point, there was a clear trend for the female diabetics to have reduced E/A ratio throughout the study (Figure 1A). In addition, the percentage of change in the E/A ratio with respect to 4 week time point was significantly higher in female diabetics (P < 0.05 vs. male diabetics at 12, 16 and 32 weeks and near significant at 8 weeks compared to male mice, Additional file 1: Figure S3A). Non-diabetic mice of both genders showed no significant changes with the age.

#### Systolic dysfunction

Changes in the systolic function were estimated by serial measurement of the LVEF. Female diabetics started showing reduction in the LVEF at 12 weeks after STZ-induced diabetes (Figure 1B, P < 0.05 vs. female non-diabetics), however, no changes were observed in male diabetics at this time point. With the progression of the disease, deterioration of LVEF was rapid in females compared to males (Figure 1B and Additional file 1: Figure S3B, P < 0.05 vs. male diabetics at 16 and 20 weeks).

Deterioration of LVEF was associated with marked dilation of the left ventricle as evidenced by rapid increase in the end systolic volume (Figure 1C and Additional file 1: Figure S3C) and thinning of anterior wall (Figure 1D & online Additional file 1: Figure S3D) in female diabetics compared to male diabetic mice (P < 0.05 vs. male diabetic at 12, 16 and 20 weeks).

#### Contractile dysfunction

**Figure 2:**
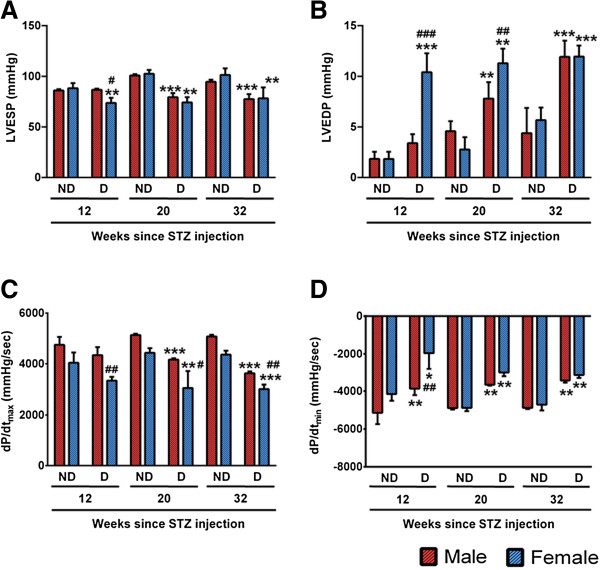
**Reduced contractility of STZ-induced female diabetic heart.** Bar graphs showing LV end systolic pressure (LVEDP, **A**), LV end diastolic pressure (LVEDP, **B**), maximum **(C)** and minimum **(D)** rates of developed pressure (dP/dt) among the study groups at 12, 20 and 32 weeks after STZ-induced diabetes (n = at least 8 at each time point). Values are means ± SD. Results of pair-wise comparison are illustrated. *P < 0.05, **P < 0.01 and ***P < 0.001 vs. non-diabetic of corresponding gender at corresponding time point. ^#^P < 0.05 and ^##^P < 0.01 vs. male diabetic at corresponding time point.

In addition to the echocardiographic measurements, a Millar catheter was used to directly measure the LV pressure before collecting samples for molecular analysis. As shown in Figure 2, there was rapid worsening of systolic and diastolic function in female diabetic as evidenced by the decrease of LV end systolic (Figure 2A) and increase of LV end diastolic pressure (Figure 2B) in female diabetics at 12 weeks after STZ-induced diabetes (P < 0.05 vs. female non-diabetic). Maximal (dP/dt_max_, Figure 2C) and minimal (dP/dt_min_, Figure 2D) indices of contractility also started to deteriorate at 12 weeks in female diabetic hearts (P < 0.05 vs. female non-diabetic), although it should be noted that dP/dt can be affected by low intracavitary pressure alone. With the progression of the disease, contractile parameters showed consistent deterioration. Male diabetic mice did not show any significant difference at 12 week time point (Figure 2). Although the contractility started to decline from 20 weeks in male diabetics (P < 0.05 vs. male non-diabetic), the rate of deterioration for all the contractile parameters was slower than in the female diabetics (P < 0.05 at 12, 20 and 32 weeks for all the (Figure 2).

These functional data not only demonstrate the early onset of cardiomyopathy in female diabetic mice, but also confirms the rapid evolution of the disease in females.

### Early downregulation of pro-survival molecular pathway in female diabetics

**Figure 3:**
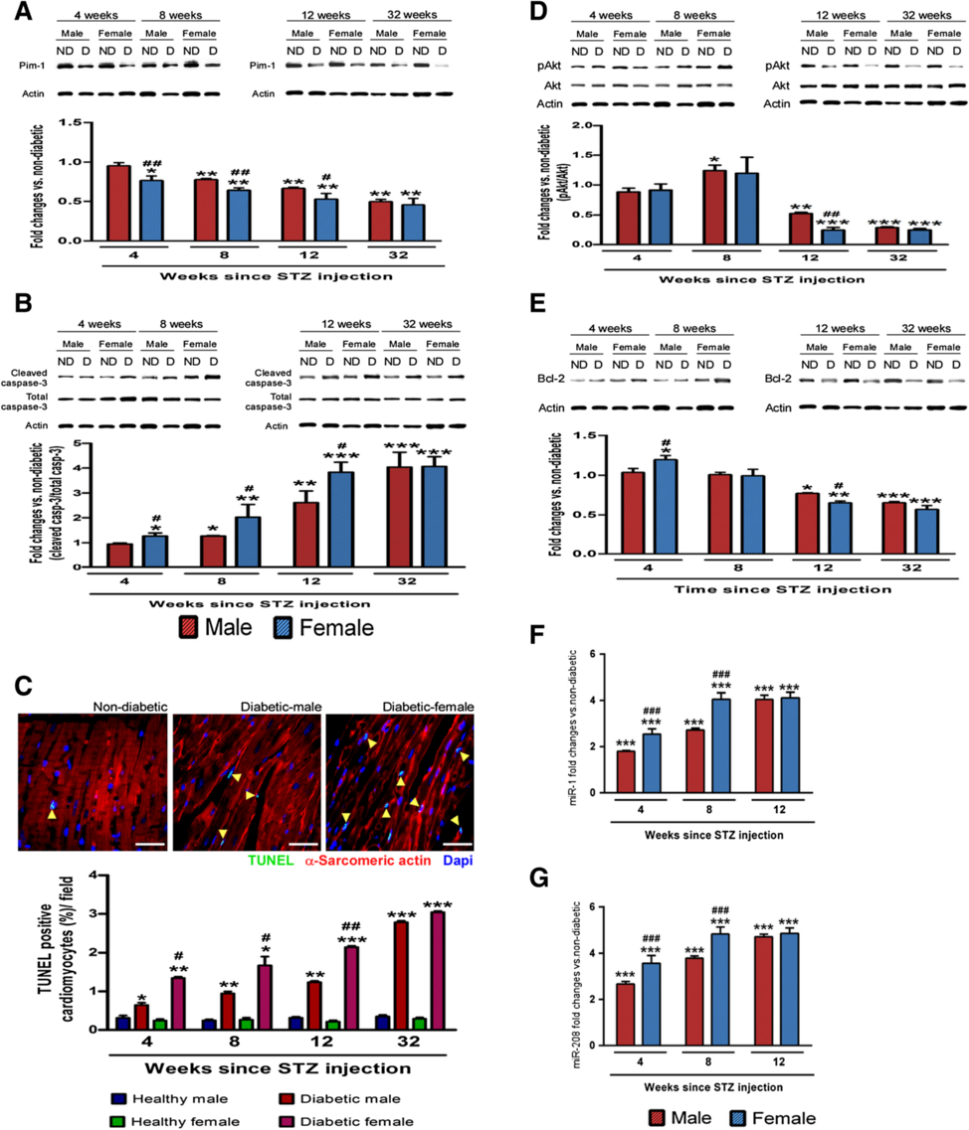
**Rapid deterioration of survival proteins and increased apoptosis in STZ-induced female diabetic heart. A-B** and **D-E** representative blots and bar graphs showing the levels of Pim-1 **(A)**, total and cleaved Caspase-3 **(B)**, pAkt/Akt **(D)** and Bcl-2 **(E)** in hearts of diabetic and non-diabetic hearts of both the genders at different time points after induction of diabetes (n = 6 at each time point). **C** representative immunofluorescence image showing TUNEL positive cardiomyocytes at 12 weeks after STZ-induced diabetes. Quantitative bar graphs showing the percentage of TUNEL positive cardiomyocytes at different time points after induction of diabetes (n = 6 at each time point). **F-G** Bar graphs showing the expression level of miR-1 **(F)** and miR-208 **(G)** in study groups (n = 5 at each time point). Values are means ± SD. Results of pair-wise comparison are illustrated. *P < 0.05, **P < 0.01 and ***P < 0.001 vs. non-diabetic of corresponding gender at corresponding time point. ^#^P < 0.05, ^##^P < 0.01 and ^###^P < 0.001 vs. male diabetic at corresponding time point.

Our earlier studies have demonstrated that molecular changes occurring early in the diabetic heart form the basis for the development of structural and functional changes at later stage [15,16]. To investigate if the key molecular changes occur earlier in female diabetics, we performed western blotting of cell survival and pro-apoptotic proteins at 4, 8, 12 and 32 weeks after STZ-induced diabetes. Results show the marked downregulation of the pro-survival protein Pim-1 (Figure 3A) in female diabetic mice as early as 4 weeks after STZ-induced diabetes, which was associated with marked upregulation of pro-apoptotic protein cleaved Caspase-3 (Figure 3B), while no significant change was observed in male diabetics at this time point (P < 0.05 vs. female non-diabetic and male diabetic). This was further confirmed by the histological analysis of the cardiac tissue, which demonstrated marked increase in the TUNEL positive cardiomyocytes in the female diabetic heart starting from 4 weeks of STZ-induced diabetes (P < 0.05 vs male diabetic at 4, 8 and 12 weeks, Figure 3C) . To understand if diabetes also increases the necrotic cell death, we measured the level of receptor interacting protein (RIP) kinase, whose activation promotes necrosis [30]. While there was no change in the RIP expression at 4, 8 and 12 weeks after STZ-induced diabetes, there was significant increases at 32 weeks, with no marked difference between male and female diabetic hearts (Additional file 1: Figure S4). These data suggest that apoptosis is the major form of cell death in the early stages of diabetic cardiomyopathy.Other pro-survival proteins such as Akt (Figure 3D) and Bcl-2 (Figure 3E) started to decrease at 12 weeks in female diabetics (P < 0.05 vs. female non-diabetic). While male diabetics also showed modulation of the cell survival proteins at 12 weeks, the changes were less profound than in female diabetics (P < 0.05, Figure 3A and B, D and E). Gender-associated differences were lost in 32 weeks old diabetic mice, which showed equal deterioration of pro-survival proteins (Figure 3A and B, D and E). These data indicate that Pim-1 precedes the deterioration of other protective factors during the development of cardiomyopathy. For this reason, we next focused on modulators of Pim-1 expression.

MicroRNAs (miRs) are recently gaining interest due to their crucial role in post-transcriptional regulation of cardiac function [31]. Literature search and online target prediction tools (*Target Scan* and *Pictar Scan*) revealed miR-1 and miR-208a as possible inhibitors of Pim-1 expression [15,17,32]. Here, we confirmed that female diabetic hearts more abundantly express miR-1 (Figure 3F) and miR-208a (Figure 3G) at 4 weeks after STZ-induced diabetes, with further increases during evolution of cardiomyopathy (P < 0.01 vs. female non-diabetic). Male diabetic hearts also show a significant upregulation of both the miRs, although the percentage change was initially less than that of female diabetic hearts (Figure 3F and G, P < 0.05). Interestingly, gender-associated differences in miR expression disappeared with the progression of the disease with both male and female diabetics showing no difference at 12 weeks after STZ-induced diabetes (Figure 3F and G).

These data suggested that activation of miRs could be the earliest modulators of the downregulation of Pim-1.

### Downregulation of Pim-1 in diabetic human hearts

**Figure 4:**
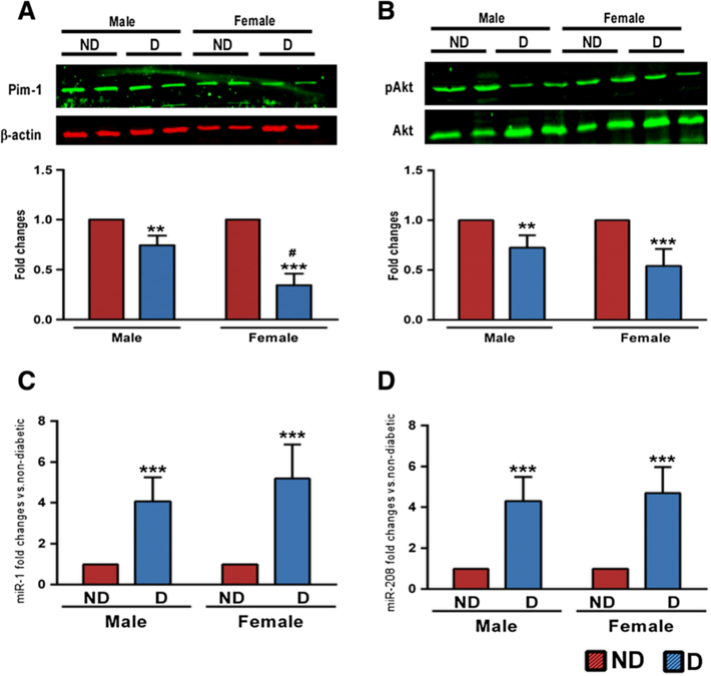
**Pim-1 downregulated in human diabetic heart. A-B** representative blots and bar graphs showing the levels of Pim-1 **(A)** and pAkt/Akt **(B)** in right atrial appendage samples collected from diabetic and non-diabetic patients undergoing on-pump coronary artery bypass graft surgery. **C-D.** Bar graphs showing the expression level of miR-1 **(C)** and miR-208a **(D)** in diabetic and non-diabetic human heart. n = at least 7 in each group. ND – non-diabetic; D – Diabetic. Values are means ± SD. **P < 0.01 and ***P < 0.001 vs. non-diabetic of corresponding gender. ^#^P < 0.05 vs. male diabetic.

To further investigate the involvement of Pim-1 in clinical settings, we measured its protein expression in the right atrial appendage samples of diabetic and non-diabetic patients undergoing coronary artery bypass graft surgery for ischemic heart disease. No difference was found in general clinical parameters among the study groups except for the level of glycosylated haemoglobin A1c (HbA1c) which was higher in the diabetic group of both genders (Additional file 1: Table S1). As shown in the Figure 4A, while both male and female diabetics demonstrate a marked decrease in the level of Pim-1, the percentage of decrease with respect to the respective non-diabetic groups was higher in females than males (Figure 4A, P < 0.05). To our knowledge, this is the first study to demonstrate the downregulation of Pim-1 in human diabetic hearts. Akt was decreased (Figure 4B) and miR-1 (Figure 4C) and miR-208 (Figure 4D) was increased in both the diabetic groups with no significant difference between genders, although there was a trend for increased levels of miR-1 in female diabetics (Figure 4C). There was no marked difference in the contractility parameter (LV ejection fraction) among the study groups, as all the patients recruited in this study had preserved ejection fraction. Although not significant, E/A ratio was at the lower level in female diabetics (Additional file 1: Table S1).

### Rescuing Pim-1 defect improves the survival of cardiomyocytes from diabetic female hearts

**Figure 5:**
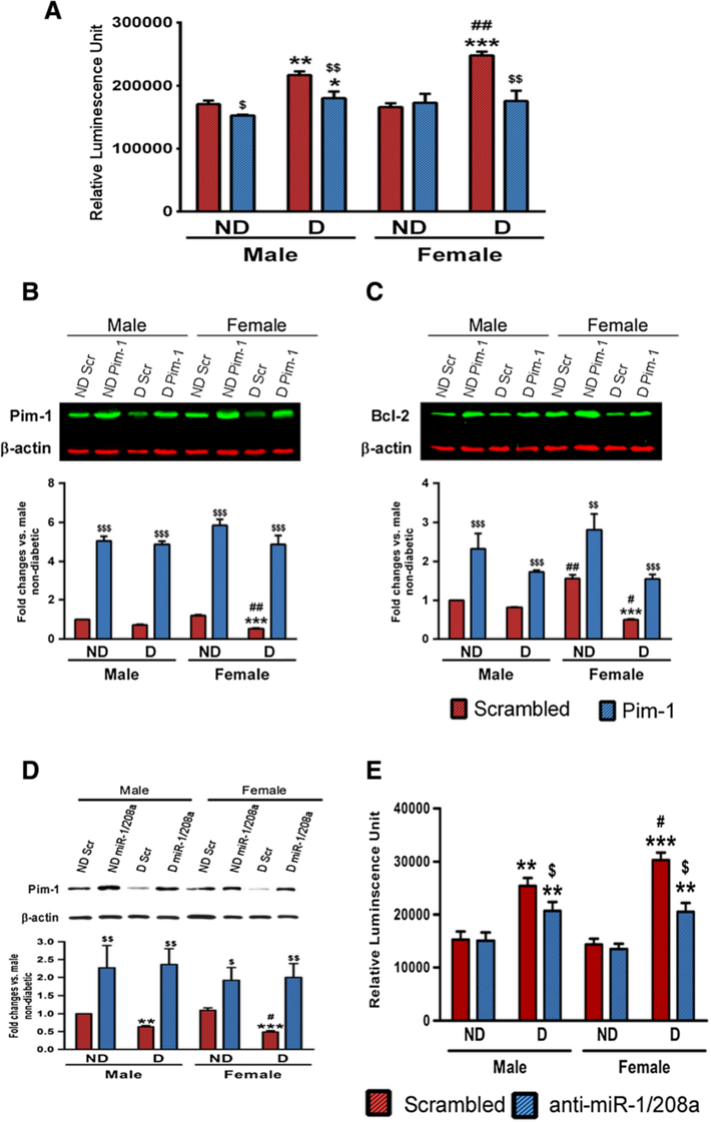
**Restoration of Pim-1 levels reverted female disadvantage. A** bar graphs showing the levels of activated caspase-3/7. **B-C** representative blots and bar graphs showing the levels of Pim-1 **(B)** and Bcl-2 **(C)** in the isolated and cultured cardiomyocytes over expressed with either Pim-1 plasmid or scrambled plasmid. **D** representative blots and bar graphs showing the levels of Pim-1 in the isolated and cultured cardiomyocytes treated with either anti-miR-1/208a or scrambled sequence. **E** bar graphs showing the levels of activated caspase-3/7. Values are means ± SD. Results of pair-wise comparison are illustrated. **P < 0.01 and ***P < 0.001 vs. non-diabetic of corresponding gender. ^#^P < 0.05 and ^##^P < 0.01 vs. male diabetic. ^$^P < 0.05, ^$$^P < 0.01 and ^$$$^P < 0.001 vs. scrambled plasmid/sequence treated group of corresponding gender. All the experiments were run in triplicate and repeated 5 independent times.

Finally, we verified if restoration of Pim-1 levels could improve the survival of female diabetic cardiomyocytes. To this aim, cardiomyocytes isolated from diabetic and non-diabetic mice at 12 weeks after STZ-induced diabetes were transfected with either hPim-1 plasmid or anti-miR-1/208a. The twelve week time point was decided due to the prominence of systolic dysfunction and significant molecular changes occurring at this time point in female diabetic mice. Caspase-3/7 activity assay showed significant increase in the level of apoptosis in both male and female diabetic cardiomyocytes (Figure 5A and E), however female diabetic cardiomyocytes showed much enhanced apoptotic activity and downregulation of Pim-1 (Figure 5B and D) and Bcl-2 (Figure 5C). Transfection with Pim-1 (Figure 5A-C) or anti-miR-1/208a (Figure 5D-E) rescued Pim-1 expression in both the male and female diabetic cardiomyocytes (Figure 5B and Figure 5D, P < 0.05 vs. scramble). Importantly, this was associated to restoration of survival signalling, i.e. Bcl-2 (Figure 5C) and decrease in the apoptotic cell death (Caspase-3/7 activity, Figure 5A&E). No change was observed in the expression levels of Akt (data not shown).

## Discussion

This study provides first molecular evidence for a gender-specific progression of diabetic cardiomyopathy. Using a mouse model, we measured functional, structural and molecular changes in the heart at different time points after STZ-induced diabetes. We found that the onset of cardiac dysfunction was more rapid and severe in diabetic females compared to males. This was associated with gender-specific LV dilation, which occurred earlier in diabetic females than males. We also demonstrate that early downregulation of pro-survival protein Pim-1 plays a major role in accelerating the progression of cardiomyopathy in female diabetics through upregulation of miR-1 and 208a. In vitro restoration of Pim-1 levels either through direct overexpression of Pim-1 or inhibition of miR-1 and 208a reverted this “*female disadvantage*” in the diabetic cardiomyocytes. Finally, molecular analysis of cardiac tissue samples from the human diabetic hearts also showed marked downregulation of Pim-1 in diabetic females.

Diastolic dysfunction is the earliest clinical sign of diabetic cardiomyopathy, followed by progression to systolic dysfunction [33]. Female diabetic mice showed marked diastolic dysfunction within 8 weeks of STZ-induced diabetes in our study, however, this was lost at 12 weeks. Diastolic dysfunction is usually associated with a decline in the E/A ratio [21], but this ratio can pseudo-normalise with progression into severe diastolic dysfunction [34]. In such cases, an increase in the end diastolic pressure can restrict the contribution of atrial contraction to LV filling [34]. Moreover, pseudo-normal or restrictive filling patterns are related to progressive LV dilation and predict cardiac death after a first MI [35]. Consistently, our echocardiography data showed LV dilation and thinning of the anterior LV wall associated with increased end diastolic pressure and decreased dP/dt_min_ in female mice at 12 weeks of STZ-induced diabetes, suggesting the exacerbation of diastolic dysfunction in female diabetic mice.

While our study demonstrated early onset of cardiomyopathy in female diabetics, Ceylan-Isik et al. reported that cardiomyocytes isolated from the female diabetic hearts were better protected than the cardiomyocytes from male diabetic hearts [36]. Similarly, cardiomyocytes isolated from young female diabetic mice by Zhang et al. exhibited normal contractile function [37]. Although these results are in contrast to our findings, the difference could be attributed mainly to the different experimental conditions. We used an *in vivo* model reflecting near clinical settings, while the above-mentioned studies used isolated cardiomyocytes. In addition, factors such as cell death and myocardial fibrosis, which play a major role in the development of cardiac dysfunction would not affect the results in *in vitro* settings.

We and others have shown that selected molecular alterations occur early in the diabetic heart which forms the basis for the development of structural changes [17,18,29]. Our earlier studies showed significant downregulation of the pro-survival protein Pim-1 in the male diabetic heart at 8 weeks after the onset of STZ-induced diabetes. Importantly, restoring the Pim-1 levels by systemic adeno-associated viral vector gene delivery halted the progression of diabetic cardiomyopathy [15]. In the current study, we found that Pim-1 was downregulated within 4 weeks of STZ-induced diabetes in the female heart, which was associated with increased pro-apoptotic caspase-3 expression. Studies on human heart samples also confirmed significant downregulation of Pim-1 in female diabetic compared to male diabetic hearts. Although it is a difficult task to translate the findings from animal studies to humans, diabetes duration for the samples collected from human diabetic heart in our study ranged between 12 and 18 years and based on the published evidence this relates to 12 to 16 weeks of diabetes duration in mice [38]. Of note, this was the time point when significant difference was observed between male and female STZ-induced diabetic mice in most of the functional and molecular parameters.

Pim-1 is a crucial component of the signalling machinery that counteracts cardiomyocyte apoptosis during the early phase of post-ischemic healing [15,17,39,40]. This was true in our study where restoration of Pim-1 increased the survival of female diabetic cardiomyocytes. Akt is the major mediator of Pim-1. Murasaki et al demonstrated marked increase in the expression levels of Pim-1 following overexpression of cardiomyocytes, while knocking down Akt reduced Pim-1 [41]. Interestingly, our results did not demonstrate any changes in the level of Akt at 4 weeks, suggesting that early implication of Pim-1 on survival could be Akt independent, but they could synergise later producing more apoptosis.

Our results newly show marked upregulation of miR-1 in the female diabetic heart. MiR-1 has been well demonstrated as the direct regulator of Pim-1 in the heart independent of Akt [17] and our earlier study showed marked improvement in the survival of male diabetic cardiomyocytes following knockdown of miR-1 [15]. In addition to miR-1, we also found early activation of miR-208a in the female diabetic mice, which might also account for increased LV dilation early in the female diabetic heart [42]. In support of this notion, inhibition of both miR-1 and -208a improved the survival of female diabetic cardiomyocytes. However, the miR expression study on human hearts did not reveal any significant difference between male and female diabetics although there was a trend for increased expression of miR-1 in female diabetics. This could be attributed to the long duration of diabetes (>12 years) when samples were collected from the patients. Another reason could be that all the patients underwent coronary artery bypass graft surgery for ischemic heart disease, in contrast to the isolated cardiomyopathy of the mouse model. Additional in vivo studies are necessary to understand the role of miR-1 and miR-208a in accelerating the development of cardiomyopathy in female diabetic hearts.

In summary, our results provide novel insights into the molecular mechanisms behind the rapid onset of cardiomyopathy in STZ-induced female diabetic mice, with preliminary data from human hearts supporting the pre-clinical study results. Future studies targeted on *in vivo* restoration of Pim-1 either by upregulation of Pim-1 or by knocking-down miR-1 will provide a platform for the development of gender specific treatment to combat the disease.

### Study limitations

In the present study we used STZ-induced type-1 diabetes model to prove our concept. Although our earlier study has demonstrated comparable disease pattern and progression in the type-1 and type-2 diabetic model [17], it is essential to demonstrate if similar gender difference exhibit in type-2 diabetes. Human data shown in this study come from right atrial appendage samples, while the results from animal study are from the ventricle. Although Lamberts et al (personal communication, 2014) showed no difference in the functional properties of the trabeculae isolated from the rat atria and ventricle, further studies will be required to confirm if the same exists in the expression pattern of Pim-1 between atria and ventricle.

## Abbreviations

EDV: End diastolic volume; ESV: End systolic volume; HbA1c: Glycosylated haemoglobin; HF: Heart failure; LVAW: Left ventricular anterior wall; LVAW_s_: Left ventricular anterior wall in systole; LVAW_d_: Left ventricular anterior wall in diastole; LVEF: Left ventricular ejection fraction; LVESP: Left ventricular end systolic pressure; LVEDP: Left ventricular end diastolic pressure; MEM: Minimal essential medium; miR: microRNA; Pim-1: Proviral integration site for moloney murine leukemia virus-1; STZ: Streptozotocin.

## Competing interests

The authors declare that they have no competing interests.

## Authors’ contributions

AM carried out the in vivo experiments, analysed the data and wrote the first draft of the manuscript; AS collected the tissue samples and carried out the western blotting; IFS maintained the animal colony and performed PCR experiments; FR carried out the in vitro experiments; PEM carried out the microRNA PCR analysis; TPR carried out the western blotting and PCR analysis; SC and MW performed human echocardiography analysis. PS collected the human tissue samples and wrote the manuscript draft; RWB and IFG were involved in human study in writing the ethics and collecting the samples during cardiac surgery; CM was involved in the in vivo experiments at late stage of the diabetes and critically reviewed the manuscript; PM co-designed the study and critically reviewed the manuscript; RK conceived and designed the study, collected the samples and wriotr the manuscript. All authors read and approved the final manuscript.

## Supplementary Material

Additional file 1 Online supplemental data. Click here for file
