# Supplementary material for: Rapid onset of cardiomyopathy in STZ-induced female diabetic mice involves the downregulation of pro-survival Pim-1
Source: Cardiovasc Diabetol. 2014 Apr 1;13:68. doi: 10.1186/1475-2840-13-68 (PMC4073808; doi:10.1186/1475-2840-13-68)

**Online supplemental data**

**Online Supplemental Table 1 - clinical characteristics of study patients**

|  | **Non-Diabetic** | | **Diabetic** | |
| --- | --- | --- | --- | --- |
|  | **Male** | **Female** | **Male** | **Female** |
| **Number of samples** | 8 | 6 | 8 | 7 |
| **Age (years)** | 61± 2 | 74± 6 | 58± 3 | 60± 2* |
| **Duration of diabetes (years)** | 0 | 0 | 14.4±4.1 | 11.5±3.5 |
| **Body Weight (kg)** | 97 ± 4 | 96 ± 9 | 100 ± 6 | 108 ± 18 |
| **Body mass index (kg/m^2)^** | 32 ±1 | 30 ± 2 | 34 ± 6 | 34 ± 4 |
| **HbA1C (mmol/mol)** | 40±12 | 36±8 | 65±16* | 59±11* |
| **LV ejection fraction** | 54±6 | 56±3 | 54±3 | 53±4 |
| **E/A ratio** | 1.25±0.14 | 1.19±0.17 | 1.09±0.25 | 0.88±0.13† |
| **Inclusion Criteria** | Elective surgery at Dunedin Hospital due to ischaemic heart disease. | | | |
| **Exclusion Criteria** | Emergency CABG surgery and additional surgery i.e. valve replacement surgery. | | | |
| Values are represented as mean ± SD; *P<0.05 vs. non-diabetic of corr esponding gender. †P=0.05 vs female non-diabetic | | | | |

**Online supplemental table 1** – showing the clinical characteristics of the study patients. All the patients underwent routine echocardiography before the surgery. Left ventricular volumes and ejection fraction were calculated from the apical 4-chamber and 2-chamber views.[[1](#_ENREF_1)] The E/A ratio was obtained in the standard way from the apical 4-chamber view, using pulsed-wave Doppler with a sample volume placed between the mitral valve leaflets in diastole.[[2](#_ENREF_2)]

**References**

1. Lang RM, Bierig M, Devereux RB, Flachskampf FA, Foster E, Pellikka PA, Picard MH, Roman MJ, Seward J, Shanewise JS *et al*: **Recommendations for chamber quantification: a report from the American Society of Echocardiography's Guidelines and Standards Committee and the Chamber Quantification Writing Group, developed in conjunction with the European Association of Echocardiography, a branch of the European Society of Cardiology**. *Journal of the American Society of Echocardiography : official publication of the American Society of Echocardiography* 2005, **18**(12):1440-1463.

2. Nagueh SF, Appleton CP, Gillebert TC, Marino PN, Oh JK, Smiseth OA, Waggoner AD, Flachskampf FA, Pellikka PA, Evangelisa A: **Recommendations for the evaluation of left ventricular diastolic function by echocardiography**. *European journal of echocardiography : the journal of the Working Group on Echocardiography of the European Society of Cardiology* 2009, **10**(2):165-193.

**Online supplemental figure Legends**

**Online supplemental figure 1:** Bar graphs showing blood glucose level at different time points after induction of diabetes. Values are mean±SD. ^**^P<0.01 vs. non-diabetic of corresponding gender at corresponding time point.

**Online supplemental figure 2:** Representative images showing the hPim-1 plasmid transfected (**A**) and non-transfected (**B**) cardiomyocytes stained with human specific Pim-1 antibody. Scale bars are 50µm.

**Online supplemental figure 3:** Line graphs showing the percent change vs. 4 weeks STZ-induced diabetes of respective gender in mitral valve flow velocity (**A**) and indexes of left ventricle (LV) function assessed (**B, C & D**) by the serial echocardiogram at different time points from STZ or STZ-vehicle (n=at least 8 at each time point). LVEF – LV ejection fraction, ESV – LV end systolic volume, LVAWs – LV anterior wall thickness during systole. Values are mean±SD. Results of pair-wise comparison are illustrated. ^*^P<0.05, **P<0.01 and ^***^P<0.001 vs. non-diabetic of corresponding gender at corresponding time point. ^#^P<0.05 and ^##^P<0.01 vs. male diabetic at corresponding time point.

**Online supplemental figure 4:** Representative blots and bar graphs showing the levels of RIP kinase in hearts of diabetic and non-diabetic hearts of both the genders at different time points after induction of diabetes (n=6 at each time point). Values are means±SD. Results of pair-wise comparison are illustrated. ^**^P<0.01 vs. non-diabetic of corresponding gender at corresponding time point.


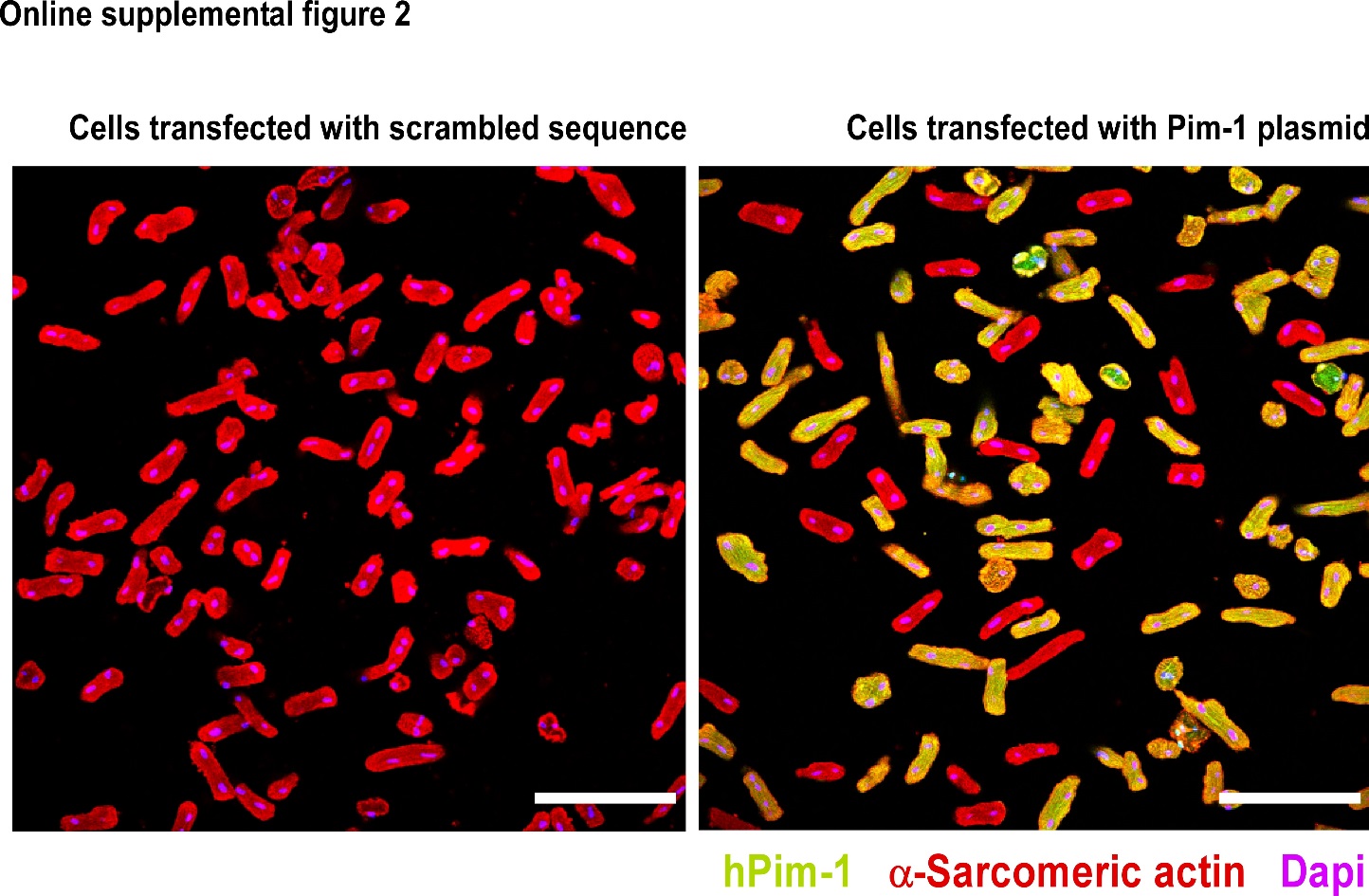

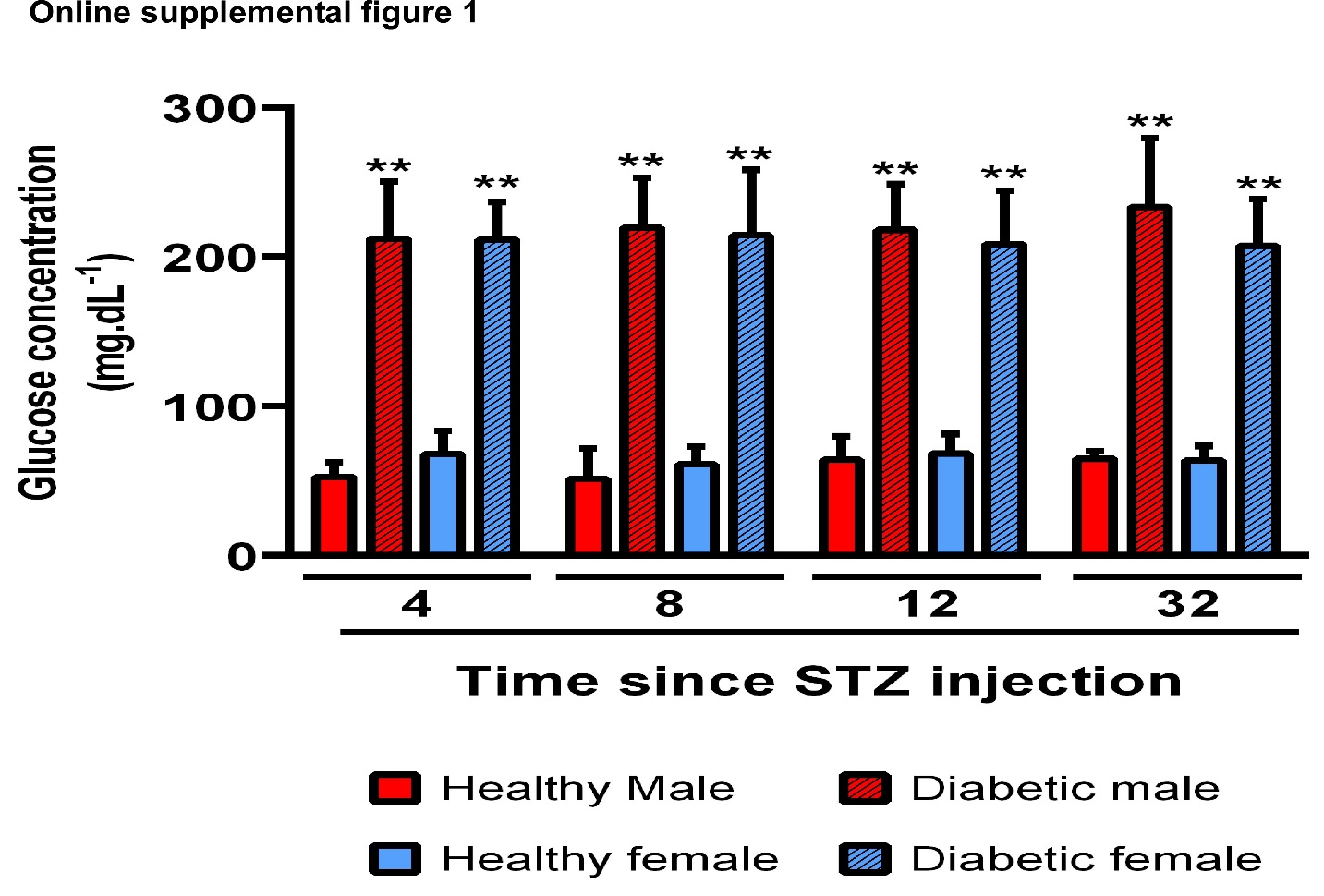


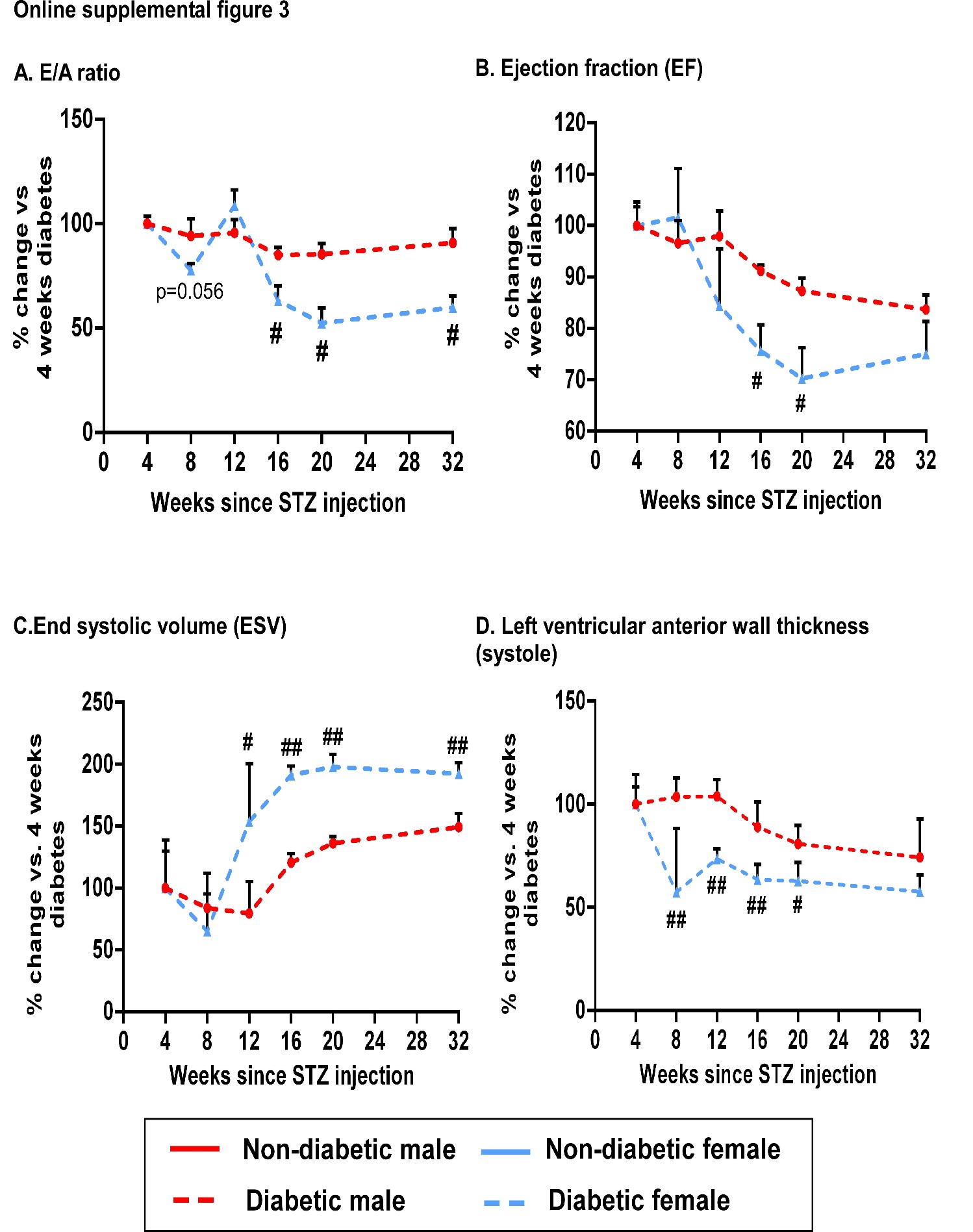


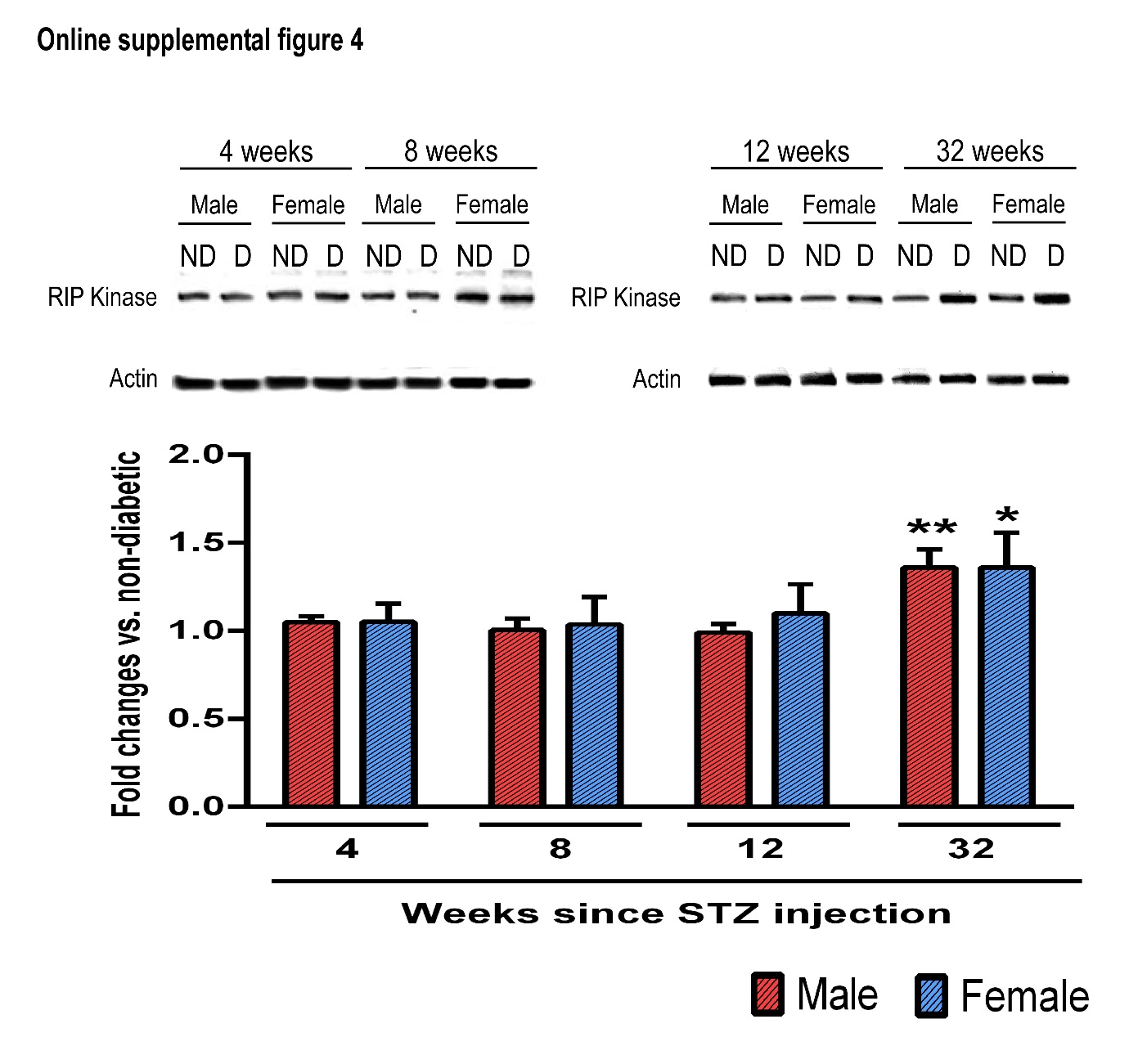

Supplement: Additional file 1 — Online supplemental data. [file 1475-2840-13-68-S1.docx]
